# New Evidence About Malignant Transformation of Endometriosis—A Systematic Review

**DOI:** 10.3390/jcm14092975

**Published:** 2025-04-25

**Authors:** Alexandra Ioannidou, Maria Sakellariou, Vaia Sarli, Periklis Panagopoulos, Nikolaos Machairiotis

**Affiliations:** Third Department of Obstetrics and Gynecology, National and Kapodistrian University of Athens Medical School, Attikon Hospital, 1 Rimini, 124 62 Athens, Greece; alexandra.ioannidou97@gmail.com (A.I.); marisakellariou@gmail.com (M.S.); vanasarli@yahoo.gr (V.S.); paninosrafaela@yahoo.gr (P.P.)

**Keywords:** endometriosis, malignant transformation, endometriosis-associated ovarian cancer (EAOC), ARID1A, predictive biomarkers

## Abstract

**Background:** Endometriosis is a benign gynecologic condition that has the risk of malignant transformation in approximately 0.5–1% of cases, most of which develop into endometriosis-associated ovarian cancers (EAOCs), such as clear cell and endometrioid adenocarcinomas. The current systematic review aims to condense recent information on the genetic and molecular mechanisms, clinical risk factors, and possible therapeutic targets of the malignant transformation of endometriosis. **Methods:** A systematic literature search of PubMed, Europe PMC, and Google Scholar was carried out according to PRISMA guidelines for articles published until December 2024. Following a screening of 44,629 titles, 43 full articles were included after meeting inclusion criteria. No case reports or reviews were included, and articles had to mention a malignant transformation of endometriosis and not just a diagnosis of cancer. The quality and risk of bias of studies were evaluated using ROBINS-I. **Results:** Malignant transformation of endometriosis is associated with genetic alterations, including ARID1A mutations, microsatellite instability, and abnormal PI3K/Akt and mTOR pathway activation. Increased oxidative stress, inflammation-driven mismatch repair deficiency, and epigenetic alterations like RUNX3 and RASSF2 hypermethylation are implicated in carcinogenesis. Clinical risk factors are advanced age (40–60 years), large ovarian endometriomas (>9 cm), postmenopausal status, and prolonged estrogen exposure. Imaging techniques like MR relaxometry and risk models based on machine learning are highly predictive for early detection. **Conclusions:** Endometriosis carcinogenesis is a multifactorial process driven by genetic changes, oxidative stress, and inflammatory mechanisms. Identification of high-risk individuals through molecular and imaging biomarkers may result in early detection and personalized therapy. Further research should aim at the development of more precise predictive models and exploration of precision medicine approaches to inhibit the emergence of EAOC.

## 1. Introduction

Endometriosis is characterized by the presence of endometrial-like tissue outside the uterus and affects approximately 5–15% of women of reproductive age. Though usually benign, about 0.5–1% of cases undergo malignant transformation, mostly into ovarian cancers, including endometrioid and clear cell adenocarcinomas [1].

Genetic mutations, most notably in the ARID1A gene, are involved in the pathogenesis of EAOC (endometriosis-associated ovarian cancer); these disrupt chromatin remodeling and activate oncogenic pathways such as PI3K/Akt [2]. Moreover, the implication of the mTOR signaling pathway in the malignant transformation of endometriosis suggests potential therapeutic targets [3].

Postmenopausal endometriosis is less frequent, but it is clinically challenging due to its malignant transformation potential. Prolonged administration of estrogen-only HRT (hormone replacement therapy) and history of definitive gynecological surgery are some of the risk factors associated with the condition [4].

This systematic review seeks to synthesize recent findings on the malignant transformation of endometriosis regarding genetic and molecular mechanisms, clinical risk factors, and potential therapeutic interventions.

## 2. Materials and Methods

This systematic review was carried out according to guidance from the PRISMA Statement. The protocol was submitted via protocols.io (https://doi.org/10.17504/protocols.io.e6nvwb2z7vmk/v1) and was accepted and published before the final search.

### 2.1. Data Sources and Search Strategy

A systematic search of three major databases—PubMed, Europe PMC, and Google Scholar—was performed with the keywords ‘’endometriosis, malignant transformation, cancer’’. All available articles were considered for this study until December 2024.

### 2.2. Eligibility Criteria for Articles’ Inclusion

**Figure 1:**
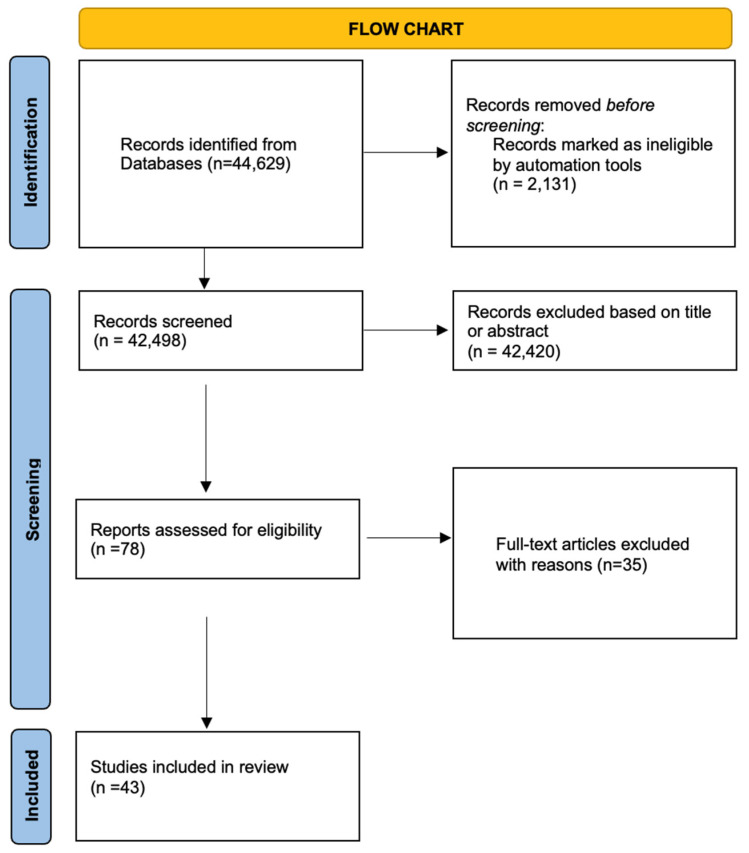
Study flowchart until final study selection.

A total of 44,629 articles were yielded by the initial database search. Two independent reviewers screened the results, excluding 2131 case reports and 42,420 articles based on their titles. Of the remaining 78 articles, 35 were excluded after applying the eligibility criteria. Articles had to be full-length research papers written in English, whereas abstracts presented at scientific meetings and review articles were excluded. In addition, only those studies were included that provided information specifically on the malignant transformation of endometriosis. This means that studies with a random diagnosis of cancer among endometriosis patients were excluded. After this strict screening, 43 articles fulfilled the eligibility criteria for inclusion in the review, as outlined in Figure 1. The risk of bias was assessed using the ROBINS-I tool, which evaluates seven domains including confounding, selection of participants, classification of exposures, deviations from intended interventions, missing data, measurement of outcomes, and selection of the reported result.

### 2.3. Data Extraction

Extracted specific data from every eligible publication consisted of date of publication, list of authors, population studied, methodologies applied, criteria related to the selection of sample type, and main outcome measures.

## 3. Results

The included studies as well as the characteristics and results of them are presented in Table 1.

### 3.1. General Observation and Characteristics

A study in 1996 found atypical endometriosis in 1.7% of cases of ovarian endometriosis (4/255), one of which developed into an endometrioid carcinoma (EC). Of 224 ovarian cancers, 24.1% (54) were related to endometriosis, and 33 of them were atypical endometriosis. Of these, 54% were clear cell carcinomas (CCCs) and 41.9% were ECs. Tumors consistent with atypical endometriosis occurred in 13 cases, which suggested its potential as a precursor lesion to clear cell and endometrioid types [5].

Another study reported ovarian endometriosis in 29% of ovarian carcinoma cases (37/127), with clear cell adenocarcinoma most frequently associated (70%). Typical endometriosis was seen in 33 cases, atypical in 29, and 25 showed both. Transitions from typical to atypical endometriosis and carcinoma occurred in 22 and 23 cases, respectively. Atypical endometriosis showed an intermediate Ki-67 index of 9.9, supporting its premalignant role, particularly for clear cell carcinomas and ECs [6].

In a series of 1000 cases of endometriosis, malignancy was found in 5.5%. Ovarian endometriosis was more often associated with malignancy (5%) than extraovarian (1%). Among malignancies, clear cell carcinomas and ECs were most common, and serous and mucinous tumors had a weaker association with endometriosis (*p* < 0.01). Malignancy arising directly in endometriosis was found in 0.9% and associated endometrial lesions in 32%, suggesting a common origin [7].

The Shizuoka Cohort Study followed 6398 women with ovarian endometriomas for 12.8 years, finding ovarian cancer in 46 (0.72%). Clear cell (39%) and endometrioid (35%) carcinomas were most common, with older age and endometrioma size ≥ 9 cm as predictive factors. These findings highlight the need for follow-up in patients with larger or postmenopausal endometriomas [8].

In a model of hyperestrogenemia and type II diabetes in rats, 6% developed atypical hyperplasia, while 4% developed EC. The development of malignancy was associated with enhanced expression of proliferative and oncogenic markers, similar to human disease, and supported the relevance of the model for studying endometriosis-associated carcinogenesis [9].

In a retrospective review of 73 clear cell ovarian cancer cases, endometriosis-associated cancers (*n* = 27) presented in younger patients (mean age 51.4 vs. 58.4 years, *p* = 0.02) and more often were unilateral (85% vs. 63%, *p* = 0.04) without ascites (0% vs. 19.5%). There were no differences in FIGO stage, histology, or 5-year survival rates, suggesting that endometriosis does not affect prognosis [10].

In another study, the malignant transformation rate of ovarian endometriosis was 1.61%. Most (82.69%) of the cases of EAOC were ovarian CCC or endometrial adenocarcinoma. Independent risk factors included age of 40–60 years; pregnancy history; tumor size; uterine myoma; and multiple foci of endometriosis. The ROC (receiver operating characteristic) analysis showed that the AUC (area under the curve) was 0.89 (*p* < 0.001), which showed a strong predictive value and thus supported early risk-based detection [11].

A cohort study regarding the incidence of ovarian cancer among 20,608 postmenopausal women with de novo or with a history of endometriosis showed that the incidence of ovarian cancer was very low, at 0.3% in the HRT and 0.5% in the controls. The application of HRT did not significantly raise the risk of ovarian cancer, except for estrogen alone, which had a higher risk (HR 2.898, *p* = 0.013). Combined estrogen–progesterone therapy and tibolone did not show an increased risk and thus are safer options for managing menopausal symptoms in this population [12].

A propensity score-matched analysis of 197,141 pairs showed that endometriosis was associated with an increased risk of endometrial cancer, HR 1.56, *p* < 0.001. Women with endometriosis had higher odds of developing an invasive endometrioid, OR 1.53, *p* = 0.005, and clear cell endometrial cancer, OR 3.0, *p* < 0.001. However, overall survival did not differ between endometriosis-associated endometrial cancer and sporadic cases [13].

In another retrospective study, among 158 patients (95 EC, 55 CCC, 8 mixed) with an average age of 57.65 years, 69% had stage I disease. EC was more frequent (60%) than CCC (35%). The mean CA125 was 559 U/mL, and tumor size was 14.12 cm, with no correlation with the stage of disease. Endometriosis was found in 67%, although only 8.9% had been previously diagnosed. Left-sided predominance was significant for EC (*p* = 0.002) but not for CCC. The overall survival rate at 1, 3 and 5 years was 85%, 78%, and 71%, respectively. Median survival time was 75 months for EC and 61 months for CCC [14].

### 3.2. Genetic and Molecular Alterations

One study analyzed 47 patients (mean age 45.5 years), 17 with ovarian cancers with endometriosis, 6 with atypical endometriosis, 17 with typical endometriosis, and 7 with a normal endometrium. p53 overexpression was found in all cases of atypical endometriosis and 82.4% of cancer cases, while only 11.8% of typical endometriosis cases showed it (*p* < 0.01). MIB1 expression was higher in cancer and atypical endometriosis but not significant (*p* = 0.073). No differences were seen in Bcl-2 or c-erb-B-2. p53 showed very high sensitivity, at 87%, and a specificity of 92% for the presence of atypical endometriosis and cancer, suggesting that it may be a marker for premalignant changes in endometriosis [15].

In another investigation of 27 ovarian endometrioid carcinoma (OECs), MSI (microsatellite instability) was demonstrated in 40.7%, including six cases of MSI-low and five of MSI-high. K-ras mutations were detected in cancerous tissues but not in normal or atypical endometriosis. The incidence of endometriosis was 44.4%, and there was no correlation between MSI and patient prognosis. Laser microdissection showed that genetic alterations were detected only in cancerous tissue, indicating that such alterations are late events in malignant transformation from atypical endometriosis to OEC [16].

Some other researchers investigated LOH (loss of heterozygosity) on 10q23.3 (D10S608), which was present in 23.5% of ovarian carcinomas compared with 4.3% of endometriotic lesions. MSI occurred frequently in both endometriosis and atypical endometriosis, in 82.6% and 75%, respectively, without significant difference. These results suggest that LOH at D10S608 may contribute to malignant transformation [17].

The contents of human ovarian cysts were analyzed, and it was found that the concentration of free iron was significantly higher in endometriotic cysts (100.9 mmol/L vs. 0.075 mmol/L, *p* < 0.01), thus increasing oxidative stress markers such as lipid peroxides and 8-OHdG (8-hydroxy-2′-deoxyguanosine). Iron concentration positively correlated with 8-OHdG (*p* < 0.01). These findings suggest that iron-induced oxidative stress may drive malignant transformation [18].

Yamamoto et al. studied PIK3CA mutations that were implicated in the development of endometriosis-associated ovarian clear cell adenocarcinoma (CCA). These authors analyzed the PIK3CA gene at exons 9 and 20 in a total of 23 CCA cases, with synchronous putative precursor lesions; each case showed endometriosis that was contiguous with carcinoma with or without cytological atypia. The authors discovered that 10 of the 23 (43%) carcinoma samples exhibited somatic PIK3CA mutations. Notably, in all mutated cases, the H1047R mutation in the kinase domain was present. These findings suggest that PIK3CA mutations, particularly the H1047R variant, are early events in the tumorigenesis of endometriosis-associated ovarian CCA [19].

Fuseya et al. evaluated the expression of MMR (mismatch repair) proteins and MSI in ovarian endometriosis and related carcinomas. The loss of MMR proteins gradually progressed from endometriosis to carcinoma. MSI-H (high-frequency microsatellite instability) was found in 14.8% of endometriosis cases and 30.4% of carcinomas. PTEN mutations and inflammatory markers were more common in cases with MSI-H. These findings suggest that inflammation-related MMR defects may play a role in the malignant transformation of ovarian endometriosis [20].

Ren et al. studied the aberrantly methylated genes associated with malignant transformation of ovarian endometriosis. In this study, the authors used methylated CpG island amplification coupled with representational difference analysis on three pairs of EAOC samples and identified nine differentially methylated candidate genes: RASSF2, RUNX3, GSTZ1, CYP2A, GBGT1, NDUFS1, SPOCK2, ADAM22 and TRIM36. Further investigation of 30 cases of EAOC revealed that RASSF2 promoter hypermethylation was significantly higher in neoplastic tissues than in ectopic endometria (*p* < 0.05), and this was accompanied by reduced RASSF2 protein expression (*p* < 0.05). These observations indicate that epigenetic inactivation through the promoter hypermethylation of RASSF2 is an early event in malignant transformation in ovarian endometriosis [21].

Matsumoto et al. studied β-catenin and PIK3CA mutations in endometriosis-associated OEC and ovarian CCC. They concluded that β-catenin mutations were present in OEC, while PIK3CA mutations are more frequently identified in ovarian CCC. These different mutation profiles indicate different molecular pathways in the development of carcinoma subtypes [22].

Worley et al. investigated the molecular alterations in endometriosis-associated ovarian CCC. The results indicated that loss of PTEN expression is an early event in the development of endometriosis, while loss of ER (estrogen receptor) and polycomb-mediated transcriptional reprogramming for pluripotency may play major roles in the malignant transformation to ovarian CCC. This study provides new insights into the pathogenic program leading to the lineage-specific transformation of endometriosis into ovarian CCC [23].

Iwabuchi et al. studied the Hb species in cyst fluid as a potential biomarker of malignancy in endometriosis. Cyst fluids obtained from 8 patients with EAOC and 35 with benign endometriotic cysts were analyzed using electronic absorption spectroscopy. The 620/580 nm absorption ratio was measured as an indirect index of the metHb/total Hb ratio. Results showed that metHb was predominant in benign cysts and that the 620/580 nm ratio had high specificity and a positive predictive value for detecting malignant transformation. These results are in line with the idea of using Hb species in cyst fluid as one of the follow-up methods for the early detection of malignancy in endometriosis [24].

Rockfield et al. studied the expression and function of NCOA4 (nuclear receptor coactivator 4) isoforms in transformed endometriotic and malignant ovarian cells. They demonstrate that mRNA levels of both NCOA4α and NCOA4β are higher in transformed endometriotic cells compared to controls. Knockdown of NCOA4 caused an increase in FTH1 (ferritin heavy chain) and p21 protein expression, reducing cell survival, whereas overexpression of NCOA4β caused a decrease in colony formation. Moreover, the expression of NCOA4α protein was higher in malignant ovarian cancer cell lines and in certain ovarian cancer subtypes compared to normal adjacent tissues. These findings suggest that NCOA4 may play a role in the progression from endometriosis to ovarian cancer [25].

Anglesio et al. in 2017 characterized deep infiltrating endometriosis lesions from 39 patients and found that 26% harbored somatic cancer driver mutations, including alterations of KRAS, PIK3CA, ARID1A and PPP2R1A genes. These mutations were confined to the epithelial compartment of the lesions. Remarkably, deep infiltrating endometriosis is generally associated with a minimal risk of malignant transformation. Results of such a nature support the concept that somatic mutations may take part in endometriosis pathogenesis, even without malignant transformation [26].

Whole-exome sequencing in another study showed that endometriotic and normal endometrial epithelia harbor recurrent somatic mutations of KRAS, PIK3CA and FBXW7 with no significant difference in the mutation burden (*p* = 0.61). Targeted sequencing revealed that the mutant allele frequencies (MAFs) of KRAS and PIK3CA were significantly higher in the endometriotic epithelium (*p* < 0.05), with some KRAS mutations exceeding 0.5 MAF, suggesting clonal expansion [27].

In another investigation, RUNX3 was found to be highly hypermethylated in both EAOC tissues and eutopic endometrium (*p* < 0.001). Silencing of RUNX3 significantly promoted ESCs (endometrial stromal cells)’ proliferation and invasion (*p* < 0.001). Estradiol enhanced RUNX3 methylation by upregulating DNMT1, driving malignancy-associated traits in a cell model. These data point toward the implication of an oestrogen–DNMT1-driven mechanism behind RUNX3 hypermethylation, driving malignant transformation related to endometriosis [28].

miRNA expression profiling, as evaluated by Szubert M et al., revealed that miR-31-3p had high expression in normal ovarian tissue, and miR-200b-3p was expressed at the lowest level in ovarian cancer tissues. miR-125b-1-3p and miR-503-5p were highly upregulated in EAOC when compared with endometriosis (*p* < 0.001). The panel of examined miRNAs effectively discriminated between normal ovarian tissue, endometriosis, and cancer but was not able to discern EAOC from HGSOC (high-grade serous ovarian cancer). Functional investigation indicated that the miRNAs affect the mTOR and RAS pathways in a manner potentially contributing to malignant transformation [29].

In a transcriptomic analysis, 2.449 protein-coding genes were found to be upregulated, while 3.131 genes were downregulated in ovarian CCC with endometriosis as compared to benign endometriomas (*p* < 0.05). Upregulated pathways included cell cycle regulation and DNA replication, whereas cytokine receptor signaling and matrisome pathways were downregulated. miRNA analysis revealed 64 upregulated and 61 downregulated miRNAs, where the most overexpressed was miR-10a-5p. The overexpression of miR-10a-5p significantly correlated with carboplatin resistance (R^2^ = 0.93), reduced proliferation rate (*p* < 0.05), and a shift toward the G1 phase (*p* < 0.0001), a finding that deserves further study. Predicted target genes of miR-10a, which include SERPINE1, CDK6 and RAP2A, were involved in proliferation and cell cycle control and were found to be downregulated [30].

Another study found that BANCR expression increased gradually during the malignant transformation of endometriosis, and the expression levels of CPNE3 were remarkably upregulated, while those of miR-612 were downregulated (*p* < 0.05). Functional experiments showed that BANCR facilitates CPNE3-mediated cell proliferation and migration, which could be repressed by miR-612 targeting both BANCR and CPNE3. In vivo models further confirmed that BANCR enhances endometrial cell survival and tumor development through this pathway. These findings suggested that BANCR may be a driving force in the malignant transformation of endometriosis and a potential biomarker and therapeutic target [31].

Additionally, the clinical and histopathological features of endometriosis and adenomyosis were studied with the aim of investigating their potential malignant transformation. The results showed neovascularization (increased expression of CD34+), high infiltration of inflammatory cells (CD3+ T-lymphocytes, CD20+ B-lymphocytes, CD68+ macrophages, and tryptase+ mast cells), and oncogenic markers, such as Ki67, p53, BCL-2, and PTEN, especially in cases with atypia and malignancy. These findings support that inflammatory, vascular, and hormonal factors drive disease progression and are responsible for symptoms like chronic pain and abnormal bleeding. Such molecular changes provide a clue to possible therapeutic targets that may help prevent disease advancement [32].

Linder et al. performed whole-exome sequencing on paired samples from women who had endometriosis surgically removed and later developed ovarian carcinoma. They found that while both endometriosis and carcinoma samples shared some somatic mutations, the specific cancer-associated mutations present in the endometriosis did not appear in the subsequent carcinomas. This suggests that ovarian carcinoma may not directly arise from endometriosis with a cancer-like genetic profile but, rather, a common precursor might be involved [33].

In another study, 7642 DEGs (differentially expressed genes) were identified in EAOC, out of which 214 genes were found to be shared with genes related to endometriosis. In addition, based on univariate Cox regression, 10 prognostic genes were identified that included ADAMTS19 (upregulated) and TUBB (downregulated). RT-qPCR and immunohistochemistry analysis confirmed their expression patterns. Additionally, a functional assay demonstrated that ADAMTS19 promoted and TUBB inhibited cell proliferation and invasion, indicating both as potential biomarkers for prognosis and therapeutic interventions in EAOC [34].

In another research from 2024, endometriosis and endometrial cancer shared 141 DEGs, associated with the JAK-STAT signaling pathway and immune pathways. PPI (protein–protein interaction) analysis identified 10 hub genes (APOE, FGF9, TIMP1, BGN, C1QB, MX1, SIGLEC1, BST2, ICAM1, and MME); among them, APOE and BGN were found to be related to prognosis. The differential expression of these genes was verified by qRT-PCR, indicating their potential as therapeutic targets [35].

### 3.3. Correlation with ARID1A/BAF250a

In an investigation about ARID1A mutations, they were found in 46% of ovarian CCCs and 30% of ECs but not in high-grade serous ovarian carcinomas. Loss of BAF250a expression was highly correlated with ARID1A mutations, suggesting its role as a tumor suppressor. Finally, ARID1A mutations were also found in atypical endometriosis adjacent to tumors but not in distant endometriotic lesions, indicating involvement in the early transformation of endometriosis into ovarian cancer [36].

Loss of BAF250a, as found from another study, was observed in 57.7% of ovarian CCCs, 38.5% of atypical endometriosis, and 19.4% of benign endometriosis; this would favor a progressive accumulation of molecular alterations. HNF-1b (hepatocyte nuclear factor) overexpression and loss of ER/PR (estrogen receptor, progesterone receptor) expression were also present in atypical endometriosis as in CCC. The data support the hypothesis of a stepwise transformation from benign endometriosis to CCC and identify the loss of BAF250a as an early event in malignant progression [37].

Research from Samartzis EP et al. declared that loss of BAF250a expression was observed in 15% of ovarian endometriomas and 5% of deep infiltrating endometriosis cases but not in peritoneal endometriosis or eutopic endometrium. Furthermore, partial clonal loss of BAF250a was observed, indicating that some of the endometriotic lesions develop early molecular changes. Significantly lower expression of BAF250a in ovarian endometriosis than in normal endometria also supported its potential as a biomarker for risk of malignant transformation [38].

Similar findings emerged from another study. Among 47 ovarian endometriotic cysts with carcinoma, ARID1A loss was present in 31 (66%) cases that involved both the carcinoma and nearby cyst epithelium but not the distant cyst epithelium. Separate endometriosis foci retained ARID1A. These findings support loss of ARID1A as an early event in tumor progression [39].

Of 179 analyzed samples in a study published in 2015, loss of BAF250a expression was detected in 22% of ECs, 47% of CCCs, 44% of contiguous endometriosis cases, and 8% of benign endometriotic ovarian cysts. pAKT (phosphorylated AKT), γH2AX, BIM, and BAX were overexpressed in EAOCs and contiguous endometriosis as compared to benign endometriosis (*p* < 0.05), while pATM, pCHK2, and Bcl2 were downregulated. γH2AX clustering in contiguous endometriosis suggested early DNA damage response activation. These findings indicate that ARID1A loss, along with (phosphatidylinositol-3-kinase) PI3K/AKT pathway activation, may be an early event in EAOC development [40].

Research from Winarto H. et al. also showed that ARID1A expression was significantly lower in endometriotic and EAOC tissues than in non-EAOC samples (*p* = 0.035), suggesting its potential role in malignant transformation. Antioxidant enzyme MnSOD (manganese superoxide dismutase) activity was also reduced in endometriotic and EAOC tissues (*p* = 0.049), while the levels of malondialdehyde (MDA), a marker of oxidative stress, were significantly elevated (*p* < 0.02), indicating an imbalance in oxidative stress regulation. While in vitro exposure to H₂O₂ demonstrated that the presence of this factor further downregulates ARID1A in endometriotic cell culture, significant effects were recorded when treated with 1000 nM of H₂O₂ (*p* < 0.05). This finding emphasizes that oxidative stress can act on ARID1A downregulation as a factor driving the progression of endometriosis toward EAOC [41].

In another investigation, a partial loss of BAF250a (ARID1A) expression was exhibited in 36% of the cases of rectovaginal deep infiltrating endometriosis (DIE), 40% of endometriosis lesions in pelvic sentinel lymph nodes (PSLNs), 30% of ovarian endometriomas, and 25% of control endometrium samples. Complete loss was not detected in any of the benign tissues. No significant associations were found between partial loss of BAF250a and hormone use (*p* = 0.106), menstrual cycle phase (*p* = 0.917), or disease stage (*p* = 0.717) [42].

Another study revealed that somatic mutations specifically in cancer-associated genes were observed in 4% of ovarian endometriosis samples, including KRAS p.G12V and PPP2R1A p.S256F along with two ARID1A nonsense mutations, p.Q403 * and p.G1926 *. Seven other cancer-related genes (BRAF, NRAS, HRAS, ERK1, ERK2, PTEN, and PIK3CA) were found negative for mutations. Interestingly, instances of co-occurrence of KRAS and ARID1A mutations were found within the single patient who had demonstrably elevated CA125 (308.4 U/mL) and history of late menarche [43].

Other researchers also found that ARID1A expression was significantly downregulated in endometrioid heterotopies compared to normal endometria, with the highest downregulation in ovarian heterotopies (3.98-fold). PgE2 (prostaglandin E2) synthase and PgE2-Rec (receptor) were also downregulated, with the highest downregulation in ovarian heterotopies (5.21-fold and 2.13-fold, respectively). The findings incriminate ARID1A, PgE2 synthase, and PgE2-Rec as potential markers for malignant transformation in endometriosis, especially in the ovarian tissue [44].

Furthermore, in a recent investigation from 2024, HDAC6 (Histone deacetylase 6) expression varied across endometriotic subtypes and correlated with ARID1A status. In ARID1A-deficient individuals, epithelial HDAC6 expression was profoundly upregulated in endometriotic lesions compared to control endometria (*p* = 0.031), particularly in ovarian endometriosis (*p* = 0.037). Deep infiltrating endometriosis exhibited the highest epithelial (*p* = 0.032) and stromal (*p* = 0.007) HDAC6 expression. These findings suggest a complex interaction between ARID1A loss and HDAC6 regulation in endometriosis, with potential implications for malignant transformation [45].

### 3.4. Other Tools for Predicting Malignancy

In a prospective study from Yoshimoto C. et al., MR relaxometry identified a close correlation between R2 values and the concentration of total iron in cyst fluid. In contrast with ovarian endometriosis (OE), EAOC demonstrated much lower R2 values and concentrations of total iron. The in vivo R2 value was used to distinguish EAOC from OE with a sensitivity of 86% and specificity of 94%. These findings suggest that MR relaxometry provides a non-invasive and accurate method for the prediction of malignant transformation in ovarian endometriosis [46].

In efforts to predict malignant transformation of endometriosis, EAOC was well predicted by a machine learning-based risk model using a gradient-boosting decision tree with an AUC (area under the curve) of 0.942, sensitivity of 86.8%, and specificity of 86.7%. The model outperformed a logistic regression model (AUC 0.891, *p* = 0.036). Validation testified to its high accuracy, suggesting its utility for early EAOC detection [47].

## 4. Discussion

The findings from this systematic review highlight the complex interplay between the genetic, molecular, and clinical factors contributing to the malignant progression of endometriosis into EAOC. An ongoing theme present in the majority of the research is the shared coexistence of endometriosis and ovarian CCC and EC, with atypical endometriosis representing a potential precursor lesion [5,6]. Increased malignancy risk was also found in women presenting with ovarian endometriomas, particularly with size >9 cm or in postmenopausal women [8].

Genetically, ARID1A mutation is among the most significant changes identified in ovarian cancers with endometriosis, and it has a very high prevalence in clear cell and endometrioid histologies [36,37]. Loss of BAF250a, a tumor suppressor protein that is associated with ARID1A, is an early event in the process of malignant transformation, seen in contiguous endometriosis lesions but not in distant foci [38,39]. Loss of other molecular changes—including PIK3CA, PTEN, KRAS—and microsatellite instability also highlight the role of dysregulated oncogenic pathways in carcinogenesis [16,20,22].

Oxidative stress has also been identified as a key inducer of malignant transformation, with increased free iron and oxidative damage markers in endometriotic cysts [18]. Epigenetic alterations, including hypermethylation of tumor suppressor genes like RUNX3 and RASSF2, also contribute to malignant transformation by suppressing key regulatory pathways [21,28]. In addition, transcriptomic research has shown evidence for differential gene expression of genes associated with inflammation, cell cycle, and immune response, suggesting a multifactorial etiology of EAOC [30,35].

Clinically, risk factors such as age (40–60 years), tumor size, and pregnancy history have been shown to make malignant transformation more likely [11]. The contribution of hormone replacement therapy (HRT) is unclear; estrogen-alone therapy appears to augment risk, while combined estrogen–progesterone therapy does not [12].

Several predictive and diagnostic models have been established to identify patients at high risk of EAOC. Imaging techniques like MR relaxometry and biomarkers like hemoglobin species in cyst fluid have been found to be very sensitive and specific in distinguishing benign from malignant conditions [24,46]. Machine learning models have also been found to be useful in early detection, with better performance compared to traditional logistic regression models in risk prediction [47].

Of course, our research has some limitations, as the focus was primarily on EAOC, with limited research on malignant transformation in extra-ovarian endometriosis. Additionally, while many studies report genetic and molecular alterations, few investigate the functional consequences of these mutations or follow patients over time to assess their actual risk of malignancy.

## 5. Conclusions

This systematic review emphasizes the multifactorial pathogenesis of endometriosis-associated malignancy, which involves genetic mutation, oxidative stress, and epigenetic alteration. The identification of the ARID1A mutation, microsatellite instability, and inflammatory mechanisms as key drivers of carcinogenesis provides valuable clues to potential diagnostic markers and targets for therapy. Beyond advancing molecular understanding, these findings underscore the need for timely and decisive clinical interventions. In select high-risk cases, especially where atypical histologic features or persistent large lesions are present, early surgical management should be strongly considered as part of a proactive strategy to mitigate malignant transformation. Given the firm link between ovarian endometriosis and EAOC, careful follow-up in high-risk patients, particularly those with atypical endometriosis or giant endometriomas, is advised.

In addition to advancing scientific understanding, these findings highlight opportunities for clinical translation, such as developing improved screening protocols, incorporating molecular biomarkers into risk stratification models, and tailoring surveillance strategies. Future research should focus on maximizing predictive models using machine learning, validating biomarker panels, and exploring targeted therapies against mTOR, PI3K/Akt, and epigenetic modulators. A deeper understanding of the stepwise progression from endometriosis to malignancy will ultimately support earlier detection and improved patient outcomes.

## Figures and Tables

**Table 1:** Studies reviewed and reported outcomes. NS: not specified, EAOC: endometriosis-associated ovarian cancer, OC: ovarian cancer, CC: clear cell, E: endometriosis, HRT: hormone replacement therapy, OCCC: ovarian clear cell carcinoma, EC: endometrioid carcinoma, HR: hazard ratio, OR: odds ratio, LOH: loss of heterozygosity, CCA: clear cell adenocarcinoma, MMR: mismatch repair, HGSOC: high-grade serous ovarian cancer, PI3K: phosphatidylinositol-3-kinase, ET: endometriotic tissue, MnSOD: manganese superoxide dismutase, ER: estrogen receptor, PSC: papillary serous carcinoma, CG: control group, PgE2: prostaglandin E2, NE: normal endometrium, BCL-2: B-cell lymphoma 2, PTEN: phosphatase and tensin homolog, OE: ovarian endometriosis.

Author	Year	Patients’ Age (Mean Age in Years)	Sample Size	Type of Malignancy	Result
Fukunaga et al. [5].	1996	45.3	255	Ovarian malignant epithelial tumors	Atypical endometriosis has precancerous potential, mainly associated with CC and EC
Ogawa et al. [6].	2000	51.4	127	OC	Atypical endometriosis is a premalignant lesion, particularly for CC and endometrioid adenocarcinomas
Stern et al. [7]	2001	NS	1000	All types of malignancy	Stronger association of endometriosis with CC and EC than serous and mucinous tumors (*p* < 0.01)
Kobayashi et al. [8].	2007	OC: 50 ± 9, without OC: 39 ± 7	6398	OC	Higher predisposition to CC and endometrioid OC; advancing age and large endometriomas are predictive factors
Wang et al. [9].	2014	8–12 weeks (rats)	90 adult female rats	Malignant transformation in rats	Endometriosis patients may suffer a higher risk of canceration than eutopic endometria
Scarfone et al. [10]	2014	Arising from E: 51.4 ± 10.0, No E: 58.4 ± 11.2	73	OCCC	Endometriosis-associated tumors had ascites less frequently and were often unilateral; endometriosis did not impact on tumor stage or prognosis in OCCC
Zhou et al. [11]	2018	EAOC: 49.57 ± 9.47, EC: 42.53 ± 10.47	208	EAOC	Malignant transformation rate of ovarian endometriosis: 1.61%. Risk factors: age, 40–60 years; pregnancy history; tumor size; uterine myoma; and multiple foci of endometriosis
Lee et al. [12]	2023	55.0 ± 4.6	20.608	OC	HRT did not significantly raise the risk for ovarian cancer, except for estrogen alone, which had a higher risk (HR 2.898, *p* = 0.013)
Farolfi et al. [13]	2024	E: 40.2 ± 11.1, Control: 40.1 ± 11.1, E-related endometrial cancer: 52.1 ± 11.4, Non-E related endometrial cancer: 51.6 ± 11.8	6,652,752	Endometrial cancer	Increased risk of endometrial cancer (HR 1.56, *p* < 0.001). Higher odds of developing invasive endometrioid (OR 1.53, *p* = 0.005), and CC endometrial cancer (OR 3.0, *p* < 0.001). Overall survival did not differ
McMullan et al. [14]	2024	57.65	158	Ovarian CCC and EC	Left-sided predominance was significant for EC (*p* = 0.002) but not for CCC
Sáinz de la Cuesta et al. [15]	2003	NS	47	EAOC	Overexpression of p53 in atypical endometriosis and cancer associated with endometriosis
Amemiya et al. [16]	2004	NS	27	Ovarian EC	K-ras mutation and microsatellite instability are late events in malignant transformation from atypical endometriosis to ovarian EC
Ali-Fehmi et al. [17]	2006	NS	32	OC	LOH at D10S608
Yamaguchi et al. [18]	2008	NS	36	OC	Free iron in endometriotic cysts was strongly associated with greater oxidative stress and frequent DNA mutations
Yamamoto et al. [19]	2011	NS	79	CCA	PIK3CA mutations, particularly the H1047R variant, are early events in the tumorigenesis of endometriosis-associated ovarian CCA
Fuseya et al. [20]	2012	NS	64	OC	Inflammation-related MMR defects may play a role
Ren et al. [21]	2014	EAOC:44.19 ± 9.86, E: 43.12 ± 4.20, NE: 43.52 ± 5.16	83	EAOC	Epigenetic inactivation through the promoter hypermethylation of RASSF2
Matsumoto et al. [22]	2015	54.1	112	Ovarian EC and OCCC	β-catenin mutations were present in ovarian EC, while PIK3CA mutations are more frequently identified in OCCC
Worley Jr. et al. [23]	2015	NS	NS	OCCC	Loss of PTEN expression is an early event in the development of endometriosis, while loss of ER and polycomb-mediated transcriptional reprogramming for pluripotency may play major roles in the malignant transformation to OCCC
Iwabuchi et al. [24]	2016	benign cysts: 40.0, EAOC: 49.5	43	EAOC	metHb was predominant in benign cysts and the 620/580 nm ratio had high specificity and a positive predictive value for detecting malignant transformation
Rockfield et al. [25]	2017	NS	100	OC	NCOA4 may play a role
Anglesio et al. [26]	2017	37	39	Gene mutations	Alterations of KRAS, PIK3CA, ARID1A, and PPP2R1A genes in deep infiltrating endometriosis
Suda et al. [27]	2018	NS	189	Tissue without cancer	Mutant allele frequencies of KRAS and PIK3CA were significantly higher in the endometriotic epithelium (*p* < 0.05)
Wang et al. [28]	2022	NS	139	EAOC	RUNX3 methylation by upregulating DNMT1
Szubert et al. [29]	2023	Endometrial cyst: 38.92 ± 13.51, EAOC: 56.80 ± 12.46, HGSOC: 64.31 ± 1.28, CG: 58.10 ± 12.52	135	EAOC, HGSOC	High expression of miR-31-3p in normal ovarian tissue and miR-200b-3p at the lowest level in ovarian cancer tissues. miR-125b-1-3p and miR-503-5p were highly upregulated in EAOC
Collins et al. [30]	2023	Benign: 30.5, Malignant: 53	35	OCCC	miR-10a-5p deserves further studies
Liu et al. [31]	2024	EOAC: 47.9 ± 4.4, E: 45.0 ± 5.2, CG:47.3 ± 6.5	52	EAOC	BANCR expression increased during the malignant transformation of endometriosis; expression levels of CPNE3 were remarkably upregulated, while those of miR-612 were downregulated (*p* < 0.05)
Istrate-Ofiteru et al. [32]	2024	NS	243	EC, CC adenocarcinoma	Neovascularization (increased expression of CD34+), high infiltration of inflammatory cells (CD3+ T-lymphocytes, CD20+ B-lymphocytes, CD68+ macrophages, and tryptase+ mast cells), and oncogenic markers, such as Ki67, p53, BCL-2, and PTEN, in cases with atypia and malignancy
Linder et al. [33]	2024	NS	NS	EAOC	Specific cancer-associated mutations present in the endometriosis did not appear in the subsequent carcinomas
Yang et al. [34]	2024	NS	247	EAOC	ADAMTS19 expression was elevated, TUBB expression was reduced in EAOC
Ma et al. [35]	2024	NS	164	Endometrial cancer	There are 10 central genes: APOE, FGF9, TIMP1, BGN, C1QB, MX1, SIGLEC1, BST2, ICAM1, MME. APOE and BGN show significant correlation
Wiegand et al. [36]	2010	NS	683	OC	ARID1A mutation and loss of BAF250a
Xiao et al. [37]	2012	NS	86	CCC and PSC	Loss of ARID1A/BAF250a expression
Samartzis EP et al. [38]	2012	NS	240	OC	Loss of ARID1A/BAF250a-expression
Ayhan et al. [39]	2012	52	47	OCCC and ovarian EC	loss of ARID1A
Chene et al. [40]	2015	NS	179	OCCC and ovarian EC	ARID1A loss, along with PI3K/AKT pathway activation, may be an early event in EAOC development
Winarto et al. [41]	2016	NS	23	EAOC	ARID1A mRNA expression and MnSOD was lower in ET and EAOC
Borrelli et al. [42]	2016	NS	90	-	Partial loss of BAF250a protein expression
Zou et al. [43]	2018	32	248	Gene mutations	Cancer-driver gene mutations in ovarian endometriosis (KRAS, PPP2R1A and ARID1A)
Dyatlova et al. [44]	2019	22–35	58	NS	ARID1A, PgE2 synthase, and PgE2-Receptor potential markers
Zingg et al. [45]	2024	Non E: 40.5, E: 34.1	241	NS	There is a complex interaction between ARID1A loss and HDAC6
Yoshimoto et al. [46]	2016	NS	82	EAOC	MR relaxometry can discriminate between EAOC and OE
Chao et al. [47]	2022	36.4 ± 8.4	6809	EAOC	Risk model for predicting EAOC

## Data Availability

Available from A.I., N.M. upon reasonable request.
